# Late transfer to seawater improves intestinal development of osmoregulatory capacity in Atlantic salmon smolts

**DOI:** 10.1007/s10695-026-01814-w

**Published:** 2026-10-14

**Authors:** Raneesha de Fonseka, Marius Takvam, Kristina Sundell, Elisabeth Jönsson, Tom Ole Nilsen, Vibeke Vikeså, Henrik Sundh

**Affiliations:** 1 Blue Food–Centre of Future Seafood, University of Gothenburg, PO Box 463, SE-405 30 Gothenburg, Sweden; 2 Department of Biological and Environmental Sciences, University of Gothenburg, PO Box 463, SE-405 30 Gothenburg, Sweden; 3 Department of Biological Sciences, University of Bergen, PO Box 7803, N-5020 Bergen, Norway; 4 NORCE, Norwegian Research Centre AS, NORCE Environment, PO Box 22, N-5838 Bergen, Norway; 5 Skretting Aquaculture Innovation, Sjøhagen 6, N-4016 Stavanger, Norway; 6 Swedish Mariculture Research Centre (SWEMARC), University of Gothenburg, PO Box 463, SE-405 30 Gothenburg, Sweden; 7 Department of Biological and Environmental Sciences, University of Gothenburg, PO Box 463, Medicinaregatan 7B, 413 90 Gothenburg, Sweden

**Keywords:** Fasting, High-lipid, Osmoregulation, Mucosa, Parr-smolt transformation

## Abstract

The development and function of intestinal fluid transport capacity in Atlantic salmon, determine seawater (SW) readiness, but poorly understood in modern aquaculture practices. This study aimed to investigate the development of intestinal fluid transport capacity during smoltification and the effect of short-term fasting and a high-lipid diet. Two identical experiments were conducted using two feed types during smoltification: (1) control and (2) high-lipid diet with extra 5% fat. Smoltification was induced by a 6-week short-day (12L:12D) photoperiod, followed by continuous light (LD 24:0) at 12 °C freshwater (FW). Fish were transferred to SW at 144, 300, and 480 degree-days (dd), and each group was further divided into fed and fasted (4 days pre-transfer). In fish fed the control diet, intestinal tissue mass, Na^+^,K^+^,-ATPase (NKA) activity, and fluid transport capacity increased with transfer time in both FW and SW, indicating that 480 dd group had the most developed hypo-osmoregulatory capacity. In all groups, fasting reduced intestinal tissue mass for at least 6 weeks in SW. Fish fed the high-lipid diet exhibited higher NKA and fluid transport capacity in FW for both 300 dd and 480 dd groups. After 6 weeks in SW, the 480 dd group showed higher intestinal mass, NKA activity, and fluid uptake than the other groups and was not affected by fasting. Overall, late SW transfer promoted intestinal growth and increased transport capacity. Fasting negatively affected intestinal fluid uptake during the early SW phase, whereas a high-lipid diet accelerated intestinal development and mitigated the negative effects of fasting.

## Introduction

The gastrointestinal tract is a key osmoregulatory tissue in marine teleosts, compensating for osmotic water loss by processing and absorbing 70–85% of the ingested seawater (SW) (Hirano et al. 1976; Grosell 2010). Intestinal fluid absorption is ion-coupled, driven by basolaterally located Na^+^, K^+^-ATPases (NKA) that generate an inward Na^+^ gradient that in turn drives apical uptake of Na^+^, Cl^−^, and K^+^ ions into the enterocytes via NKCC2 and NCC transporters (Musch et al. 1982; Sundh et al. 2014; Gaetano et al. 2025). In addition, Na^+^ ions are absorbed in co-transport with nutrients (Ferraris and Ahearn 1984; Sundell and Ronnestad 2011; Hedén et al. 2022) while Cl^−^ ions enter the enterocytes via the anion exchanger, Cl^−^/HCO_3_ (Slc26a6). Increased luminal HCO_3_^−^ promotes the precipitation of calcium carbonate (CaCO_3_) and magnesium carbonate (MgCO_3_) and reduces the luminal osmolality and thereby facilitates water absorption (Walsh et al. 1991; Wilson and Grosell 2003). The net ion absorption mechanism further creates a hyperosmotic microenvironment in the lateral intercellular space (LIS), allowing passive water absorption along the osmotic gradient (Grosell 2010; Sundell and Sundh 2012). The ion transport across the intestinal epithelium is therefore critical for water absorption in the marine environment (Marshall and Grosell 2006; Grosell 2010).

During migration from freshwater (FW) to SW, Atlantic salmon maintain osmotic balance by elevated drinking rates and increased intestinal fluid absorption as described above (Usher et al. 1988; Fuentes et al. 1996; Sundell and Sundh 2012). During parr-smolt transformation, the intestine undergoes a preparatory functional remodelling where a gradual increase in NKA enzyme activity and fluid transport capacity in the intestine is observed (McCormick and Saunders 1987; Veillette et al. 1993, 1995; Sundell et al. 2003). These smoltification-induced changes require energy, reflected by a concurrent increase in lipid metabolism and lipase activity (Woo et al. 1978; Sheridan et al. 1985, 1989). Thus, a FW diet with higher lipid levels may support the higher energy demand during the parr-smolt transformation. Indeed, a high-lipid diet containing 5% additional fat during smoltification has been shown to accelerate NKA activity in key osmoregulatory organs and mitigate the negative effects of short-term fasting on subsequent growth performance (de Fonseka et al. 2026). However, the influence of a high-lipid diet on intestinal transport capacity in smolts and its consequences for subsequent SW performance remain unknown.

Commercial smolt production relies on photoperiod manipulation to induce smoltification and typically employs a square wave photoperiod (winter and spring signal) (Duston and Saunders 1990; Björnsson et al. 1995; Sigholt et al. 1995; Duncan and Bromage 1998). Although production protocols have been continuously refined to improve smolt quality and increase production efficiency (Strand et al. 2018; Striberny et al. 2021; Fraser et al. 2023) the Atlantic salmon farming industry continues to experience substantial production losses following SW transfer, with suboptimal smolt quality at the time of SW transfer identified as a main contributing factor (Bang Jensen et al. 2020; Oliveira et al. 2021; Moldal et al. 2026). Gill NKA activity and gene expression are the primary indicators used to assess smoltification status and to determine the time of SW transfer (Zaugg and McLain 1976; McCormick 2012). Based on these indicators, the common recommendation for transfer is between 300 and 400 dd (Stefansson et al. 1998; Handeland et al. 2004). However, the intestine, an equally important osmoregulatory organ as gills, has received comparatively less attention in commercial practices of determining SW transfer time. Insufficient intestinal osmoregulatory development at SW transfer would contribute to osmoregulatory stress which could contribute to increased disease susceptibility, reduced growth and increased mortality during the first period after SW transfer (Vargas-Chacoff et al. 2018). Recent studies have suggested that peak intestinal NKA activity may occur later than in the gills (Vargas-Lagos et al. 2018; Takvam et al. 2024; de Fonseka et al. 2026). Consequently, the intestinal osmoregulatory capacity may not be fully developed at the currently recommended time of SW transfer (Vargas-Lagos et al. 2018; Silva-Marrero et al. 2025; de Fonseka et al. 2026) Therefore, it is important to address knowledge gaps regarding the development of intestinal fluid transport capacity in relation to timing of SW water transfer in photoperiod-induced smolts.

Fasting is routinely applied in Atlantic salmon farms before transport of smolts from FW to SW. Typically lasting 3–4 days, fasting reduces the production of metabolic waste and oxygen demand during transport (Southgate 2008; Waagbø et al. 2017). As the intestine is metabolically costly to maintain, fasting-induced intestinal tissue regression represents an energy-saving strategy in many vertebrates (German et al. 2010; Zhou et al. 2022). In fish, fasting reduces intestinal mass, digestive enzyme activity, and induces morphological changes (Krogdahl and Bakke-McKellep 2005; Zaldúa and Naya 2014). While fasting-related structural changes have been widely studied, little is known about how fasting before SW transfer affects the intestinal fluid uptake capacity at transfer and during the first weeks in SW.

The aim of this study was to gain knowledge about the development of the overall intestinal fluid transport capacity and osmoregulatory function in Atlantic salmon during smoltification and subsequent SW transfer in aquaculture setting. The objectives were (1) to assess intestinal tissue mass, intestinal fluid transport capacity, and NKA activity at different times following SW transfer; (2) to examine the effects of short-term fasting prior to SW transfer on the development of intestinal osmoregulatory capacity; and (3) to investigate the influence of a high-lipid diet during smoltification on subsequent intestinal osmoregulatory capacity in SW. The present study was based on the same experiment previously reported by de Fonseka et al. (2026). While the previous publication focused on synchronisation of Na^+^, K^+^-ATPase activity in key osmoregulatory organs and fish growth responses, the present manuscript is specifically looking at the physiological mechanisms underlying intestinal development during smoltification and the early SW period.

## Methodology

### Ethics

The experiment was performed in accordance with the guidelines of the Norwegian Animal Welfare Act (LOV-2009–06–19–97) and the EU Laboratory Animal Directive (2010/63).

### Fish stock and rearing

The experiment was conducted at the Lerang Research Station, Forsand, Norway (Skretting Aquaculture Innovation), using all-female crossbred Atlantic salmon (Benchmark Genetics Salten-AS). The experiment contained 36 tanks (1 m diameter, 450 L) connected to an FW flow-through system to accommodate 2700 parr (∼ 45 g), and fish were randomly assigned to tanks. Smoltification was induced by a standard “square wave” photoperiod protocol (Stefansson et al. 1991), where fish were exposed to a 6-week winter signal (short-day, LD 12:12) with 12 h of light from 0800 to 2000 and 12 h of dark from 2000 to 0800 followed by a spring signal of continuous light (long-day, LD 24:0). The experimental conditions started on 6th January 2023 (day 0) as the photoperiod was set to continuous light, and the temperature was maintained at 12 °C. Water quality parameters were continuously monitored and maintained as described in de Fonseka et al. (2026).

### Experimental design

#### Experiment 1: fasting and SW transfer time

**Fig. 1:**
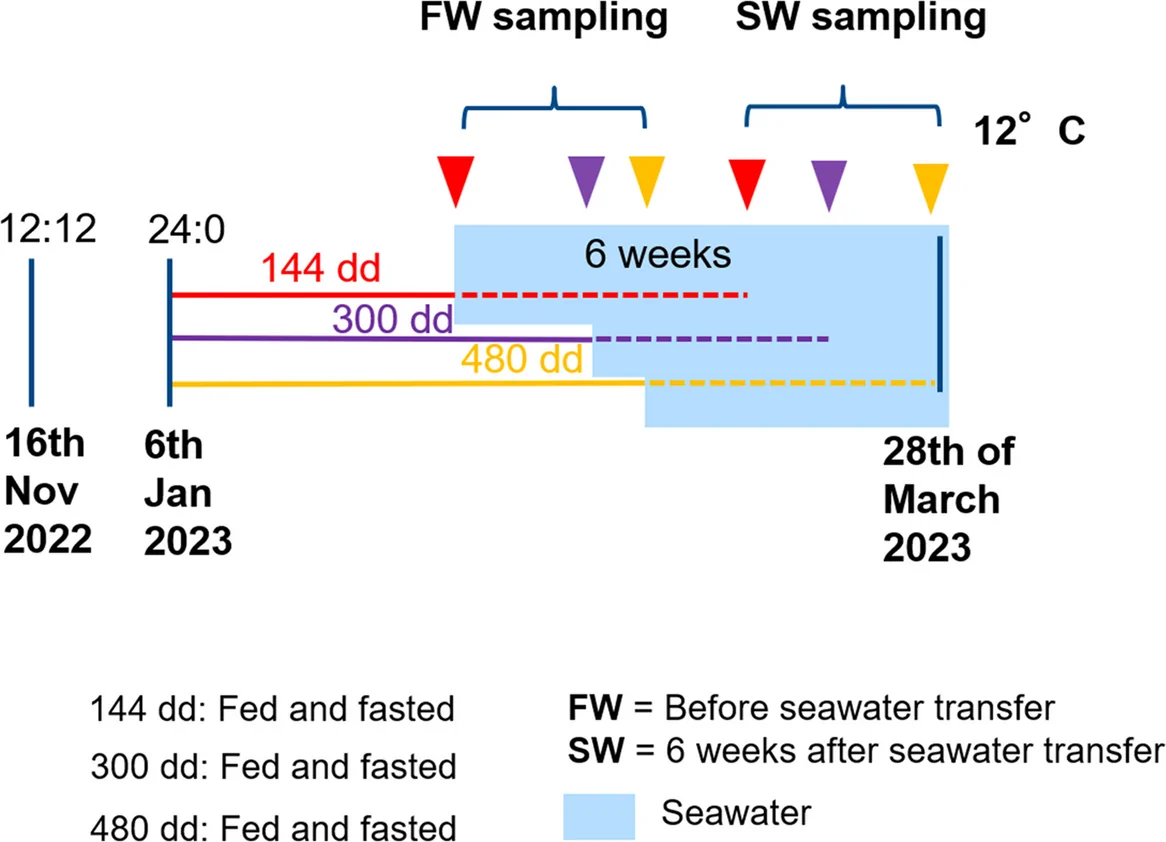
Schematic representation of the common experimental timeline for experiments 1 and 2. Each identical experiment consisted of 18 tanks at the start of the FW phase. Three transfer groups were included (144 dd, 300 dd, and 480 dd). Each transfer group consisted two sub-groups; fed and fasted (4 days before SW transfer), with triplicate tanks per sub-group. Fish were transferred to SW at the respective transfer times and remained in SW for 6 weeks before the final sampling point. Water temperature was maintained at 12 °C throughout the experiment

Experiment 1 comprised 18 tanks (*n* = 75 fish, 8.5 kg/m^3^), with three different SW transfer times (Fig. 1). Within each transfer time, fish were assigned to 2 feeding treatments: fed or fasted. Fish in 3 tanks were fed continuously, whereas those in the other 3 tanks were fasted for 4 days before the SW transfer. Fish were transferred to SW in batches (6 tanks per batch): the first batch at 144 dd, the second at 300 dd, and the third at 480 dd that produced 3 separate SW transfer times. SW transfer protocols were initiated by switching the inlet water from FW to full-strength SW in the same tank. Fish were kept in SW for 6 weeks.

In experiment 1, fish were fed with a FW control diet with two different pellet sizes (2 and 3 mm) from the start of LD 12:12 to the end of the FW stage (Table 1 in de Fonseka et al. 2026). Thereafter, a control SW diet (3 mm) was used. All the diets were produced at Skretting Aquaculture Innovation feed technology plant (Stavanger, Norway). Feeding was done using belt feeders and feeding occur 3 times per day at 0700–0759, 1030–1100, and 1400–1500 h with daily rations of at least 1% of body weight and 15% overfeeding. Feed waste was collected from each tank daily at 07:30 h and 14:30 h.

#### Experiment 2: fasting after feeding a high-lipid diet and SW transfer time

A second experiment was conducted in parallel with experiment 1, using the same experimental design and 18 additional identical tanks (*n* = 75 fish, 8.5 kg/m^3^). In contrast to experiment 1, fish received a high-lipid diet containing 5% additional fat during the FW stage. Feeding schedules for the experiment and 1 and 2 were identical.

### Sampling

Sampling took place at three separate time points per transfer group: (I) during the *parr* stage at the end of the LD 12:12 (0 dd); (II) in *FW*—shortly before the SW transfer at each transfer time (144 dd, 300 dd, and 480 dd); and (III) as *post-smolts* after 6 weeks in SW for all transfer groups (Fig. 1). Transfer time was expressed as accumulated degree-days (mean temperature × number of days), as cumulative thermal exposure has been shown to influence the completion of parr-smolt transformation (Sigholt et al. 1998).

Fish were euthanized with an overdose of anaesthetic (Tricaine PHARMAQ–TPQ, 40 mg Tricaine/L of water). For assessment of intestinal NKA activity, 4 fish from each triplicate tank, per feeding status (*n* = 12 fed and 12 fasted), and transfer time were sampled. The fish were then opened and dissected to expose the intestine which was divided into proximal (from the last pyloric caeca to the ileorectal valve) and distal (from the ileorectal valve to the anus) intestinal regions. Mucosal scrapes from the two regions were collected and stored in ice-cold intestinal SEIGE buffer (300 mM sucrose, 45 mM EDTA, 50 mM imidazole, 200 mM glycine, 50 mM ethylene glycol-bis (β-aminoethyl ether)-N,N,N′,N′-tetraacetic acid [EGTA] containing Complete™ protease inhibitor cocktail (04693124001 Roche) and frozen on dry ice for no longer than 48 h before analyses.

To assess the intestinal fluid transport capacity, an additional 3 fish from each triplicate tank, per feeding status (*n* = 9 fed and 9 fasted) were sampled. The fish were opened, and the intestine dissected and stripped of fat and large blood vessels as described by Collie and Bern 1982; Veillette et al. 1993; Sundell et al. 2003). The entire intestine, from the last pyloric caeca to the rectum, was used. The faeces were removed by gentle pressing, followed by rinsing with ice-cold, glucose-free mucosal Ringer’s. Mucosal and serosal Ringer solutions were prepared according to the fish rearing salinity. For FW fish, mucosal Ringer contained 10.0 mM mannitol, 140.0 mM NaCl, 2.5 mM KCl, 1.5 mM CaCl_2_, 0.8 mM MgSO4 *7H_2_O, and 20.0 mM glutamine, whereas the SW Ringer contained 10.0 mM mannitol, 150.0 mM NaCl, 2.5 mM KCl, 2.5 mM CaCl_2_, 1.0 mM MgCl_2_*6H_2_O_,_ 20.0 mM glutamine. Corresponding serosal Ringer solutions were prepared separately using the same base composition, except that 10.0 mM glucose was included instead of mannitol. Mannitol was used in the mucosal Ringer to reduce fluid transport through the SGLT-1 (Madsen et al. 2011). Both Ringers contained 5 mM HEPES buffer and were continuously bubbled with air, and pH was set to 7.8 with 1.5 mM TRIS base. After cleaning, intestinal weight was measured to determine intestinal tissue mass (wet weight in mg), and the tissue was stored on ice-cold mucosal Ringer’s until gut sac preparation.

At the last sampling point, post-smolts after 6 weeks in SW, an additional 4 fish from triplicate tanks and feeding status (*n* = 12 fed, 12 fasted) were used for electrophysiological analyses in Ussing chambers. For the 480 dd transfer group, the number of samples was reduced due to time constraints; therefore, only 3 fish per tank (*n* = 9 fed, 9 fasted) were used. Proximal and distal intestinal regions were analysed separately and collected similarly as described for NKA activity but left intact after they were opened longitudinally and rinsed. The tissue samples were stored in ice-cold Ringer’s until mounting in the Ussing chambers.

### Fish survival rate

$${Survival}\left(\%\right)=\frac{FE}{FI}\times 100$$

To calculate survival, the number of fish at the start (FI) and at the end (FE) of the experiment was recorded. Other growth parameters including body weight and weight gain from both experiment 1 and 2 have been reported previously in de Fonseka et al. (2026).

### Na^+^, K^+^-ATPase (NKA) activity in intestine

The intestinal NKA activity was determined using the micro-assay method of McCormick 1993), modified for the fish intestine by Sundell et al. (2003) and standardized by Takvam et al. (2024). An intestinal mucosal scrape was transferred to a sampling tube containing 100 μL of intestinal SEIGE buffer and 25 μL of SEID buffer, homogenized, and centrifuged (8000 g at 4 °C for 4 min). The retrieved supernatant was used for the NKA assay and for protein concentration measurement.

### The non-everted gut sac method for intestinal fluid uptake

**Fig. 2:**
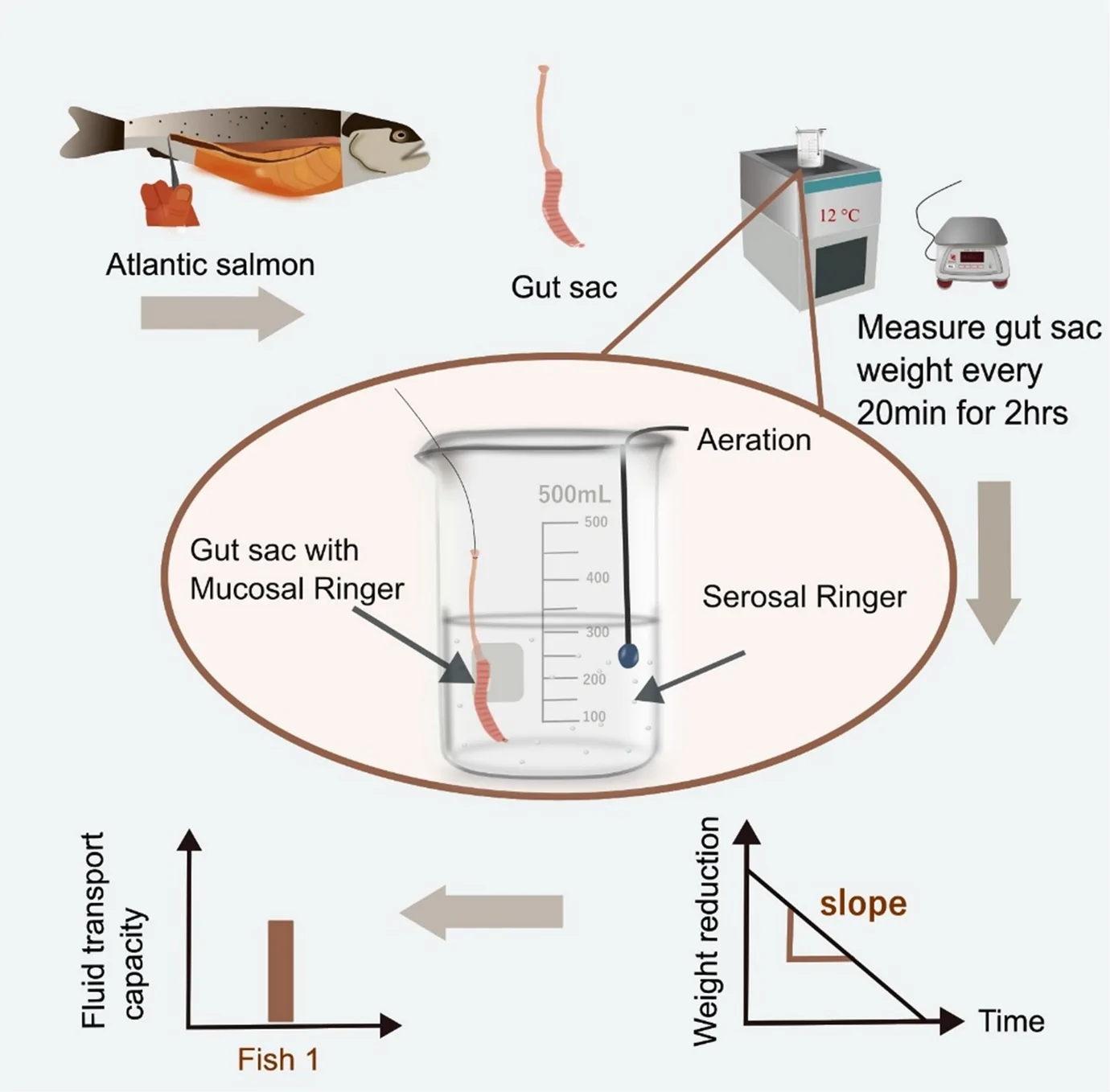
Schematic overview of the non-everted gut sac method showing the experimental workflow and key procedural steps (illustration created by the first author)

The non-everted gut sac method, as described by Veillette et al. (1993), was used to investigate intestinal fluid transport capacity in two ways: (I) for the entire intestine “per intestine” and (II) per unit area of the intestine “per area” (Jv). The excised whole intestine was first weighed to determine intestinal tissue mass, then tied at the rear end, filled with ice-cold, mucosal Ringer solution, and again tied at the proximal end, just past the last pyloric caeca, to form a sac. The filled gut sac was placed in a beaker containing continuously aerated serosal Ringer’s solution and was then incubated in a water bath at 12 °C for at least 20 min before the initial weight was recorded (Fig. 2). Subsequent weight measurements were taken every 20 min for 2 h after a gentle blotting of the sac. After the corresponding 2-h period, each gut sac was opened longitudinally to trace its outline and was digitized in ImageJ (version 1.8.0) using Java to determine its surface area. This measurement represents the serosal area as it does not account for mucosal folds and microvilli. The fluid transport capacity was calculated from the slope of the regression line for sac weight reduction (net mucosa to serosa water flux) as the fluid transport capacity “per intestine” per hour (µl h^−1^) (I). Then, fluid transport rate per intestine was normalized to the serosal surface area of the sac to achieve Jv (µl cm^−2^ h^−1^) (II). Due to a technical error, fluid transport capacity in FW was not analysed for the 480 dd transfer group in Experiment 1.

### Intestinal somatic index (ISI)

The ISI was calculated for the fish used in the non-everted gut sac method after 6 weeks in SW, in both Experiments 1 and 2, using the following equation:

$$ISI\;\left(\%\right)=\frac{Intestinal\;tissue\;mass\;(g)}{Body\;mass\;(g)}\times100$$

### Ussing chamber technique

Intestinal barrier and transport functions were determined using the Ussing chamber technique described by Sundell et al. (2003) and modified by Sundell and Sundh (2012). Proximal and distal intestines were mounted into Ussing chambers with each half chamber (area of exposure 0.75 cm^2^) filled with 4 ml Ringer’s solution for SW (150.0 mM NaCl, 2.5 mM KCl, 2.5 mM CaCl_2_, 1.0 mM MgCl_2_*6H_2_O_,_ 20.0 mM glutamine, 10.0 mM glucose, 5 mM HEPES buffer, pH 7.8). Aeration was continuously supplied to mix the Ringer’s solution within the chambers and to oxygenate the tissue. Subsequently, tissue acclimatization and stabilization of electrical parameters were ensured. Analyses of electrical parameters were carried out as described in detail by Sundh et al (2010). In brief, transepithelial resistance (TER), short-circuit current (SCC), and transepithelial potential (TEP) were measured at 5-min intervals throughout the 90-min experimental period.

After the acclimation, the SW Ringer’s solution in the serosal side of the chamber was replaced by fresh SW Ringer’s solution. At the same time, the mucosal side was replaced by Ringer’s containing hydrophilic marker ^14^C-mannitol (specific activity, 0.01 MBq/mL, Moravek Biochemicals, Brea, USA) and ^3^H- L-lysine (specific activity, 17.2 MBq/mmol, Moravek Biochemicals, Brea, USA) to assess the apparent paracellular permeability (P_app_) and active amino acid uptake dynamics, respectively. To measure the accumulated radioactivity over time, 100 µl of the Ringer solution was withdrawn from both chambers at *t* = 0 initially, and then 100 µl of the Ringer’s solution was withdrawn only from the serosal chamber at *t* = 20, 25, 30, 60, 80, 85, and 90 min. At each time point, the withdrawn volume from the serosal chamber was replaced by fresh Ringer’s solution to maintain equal volumes in each half chamber. Samples were analysed after adding 4.5 mL of scintillation fluid (Ultima Gold, PerkinElmer, Waltham, MA, USA) using a β-counter (Beckman wallac1409 Liquid Scintillation Counter, Beckman, Fullerton, CA, USA). L-lysine uptake was expressed in mol*min^−1^ cm^−2^ while P_app_ was calculated as mannitol accumulation in the serosal chamber in cm*s^−^.^1^

L-lysine uptake across the epithelium was calculated using Eq. (1)

1 $$\mathrm{L}-\mathrm{l}\mathrm{y}\mathrm{s}\mathrm{i}\mathrm{n}\mathrm{e} \mathrm\,{u}\mathrm{p}\mathrm{t}\mathrm{a}\mathrm{k}\mathrm{e} =\frac{dQ}{dt}\times \frac{1}{A}$$

where;

*dQ*/*dt*- > Accumulation of L-lysine (mol) over time (min)

*A*- > Area of the intestinal surface exposed to the chamber, 0.75 cm^2^

The apparent permeability coefficient (*P*_*app*_) for.^14^C-mannitol using the Eq. (2)

2 $${P}_{app}=\frac{dQ}{dt}\times \frac{1}{A{C}_{0}}$$

where;

*dQ*/*dt*- > Accumulation of mannitol (mol) in the serosal chamber over time (s)

*A*- > Area of the intestinal surface exposed to the chamber, 0.75 cm^2^

*C*_0_- > Initial concentration of ^14^C-mannitol on the mucosal side (mol/ml)

### Statistical analysis

Normal distribution of the data was assessed using the Shapiro–Wilk test, followed by the visual inspection of Q-Q plots, while Levene’s test was used to determine the homogeneity of variance. When assumptions were violated, a log_10_ or a square root transformation was used. Data were analysed using linear mixed-effect model to account for the hierarchical experimental design in which multiple fish were sampled from the same tank. Transfer time, fasting, and their interaction were used as fixed factors and tank number as a random factor. For parameters that show no significant tank effect (*p* < 0.05), the model was simplified to a 2-way ANOVA including transfer time and fasting as fixed factors. The same statistical approach was performed separately for each diet because the control and high-lipid diets were used during the smoltification phase in FW only, whereas all fish received the same diet following SW transfer. Consequently, diet was not included as a fixed factor in a single combined model across FW and SW phases. Effects of diet were performed by discussing the difference in response pattern for each parameter between the experiments.

To test the significant main effects of SW transfer time, fasting, and their interaction, a significance level of *α* = 0.05 was used for all parameters. Significant results were followed by a pairwise Tukey post hoc test adjusted for multiple comparisons (*p* < 0.05). Pearson correlation analysis was performed separately for control and high-lipid diet-fed fish to determine the relationship between intestinal tissue mass and intestinal fluid transport capacity per intestine in both FW and SW (*p* < 0.05). These correlation analyses were intended to describe the association within each experiment. Nonparametric tests, including the Kruskal–Wallis and Mann–Whitney *U* tests, were used when ANOVA assumptions were violated. Summary statistics are presented as mean ± SEM. Statistical analyses were performed using SPSS (version 29.0.0), and graphs were generated using GraphPad Prism (version 10.6.1).

## Results

### Experiment 1—fasting and SW transfer time

#### NKA activity

**Fig. 3:**
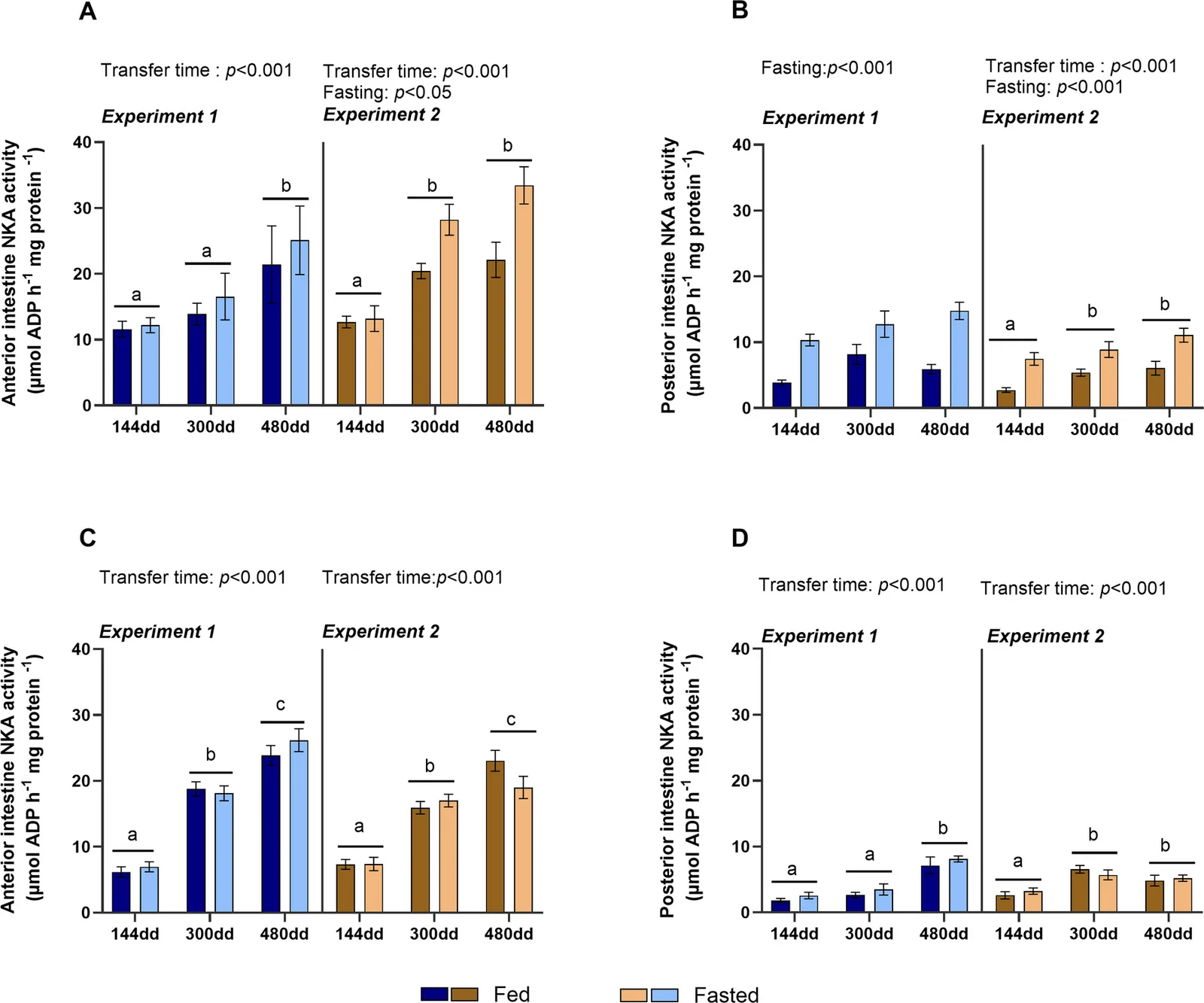
Na⁺,K⁺-ATPase activity (NKA) is presented for both the anterior and posterior intestine during smoltification in FW (**A** and **B**) and in post-smolts (**C** and **D**). Experiment 1 denotes fish fed a control diet (blue bars) during smoltification, whereas Experiment 2 denotes fish fed a high-lipid diet (brown bars) during smoltification. Both (**C** and **D**) show NKA activity in post-smolts fed the same diet in SW, with colours indicating their dietary history during smoltification: control diet (blue) and high-lipid diet (brown). Results are shown for the different transfer groups, with fed and fasted groups indicated in the legend. Different lowercase letters indicate significant differences between transfer times. For transfer time × fasting interactions, asterisks (*) denote significant differences between fed and fasted subgroups within the same transfer group. Results are presented as mean ± SEM. (*p < 0.05, ** *p* < 0.01, *** *p* < 0.001) and *n* = 12 fed and *n* = 12 fasted fish per transfer time were used (reprinted from de Fonseka et al. 2026 Figs. 5C, D and 6C, D, Copyright 2026.® with permission from Elsevier)

In the anterior intestine, higher NKA activity was observed in the 480 dd transfer group compared to other transfer groups in FW (*p* < 0.001, Fig. 3A). NKA activity in the posterior intestine did not significantly differ among transfer groups in FW (Fig. 3B). In FW, fasting did not significantly influence the NKA activity in the anterior intestine (Fig. 3A). However, NKA activity in the posterior intestine was generally higher in the fasted fish compared to the fed fish (*p* < 0.001, Fig. 3B).

SW post-smolts in the 480 dd transfer group had higher NKA activity in both anterior and posterior intestines compared to the other two transfer groups (*p* < 0.001, Fig. 3C, D). Fasting did not significantly influence the NKA activity in post-smolts.

#### Intestinal tissue mass

**Fig. 4:**
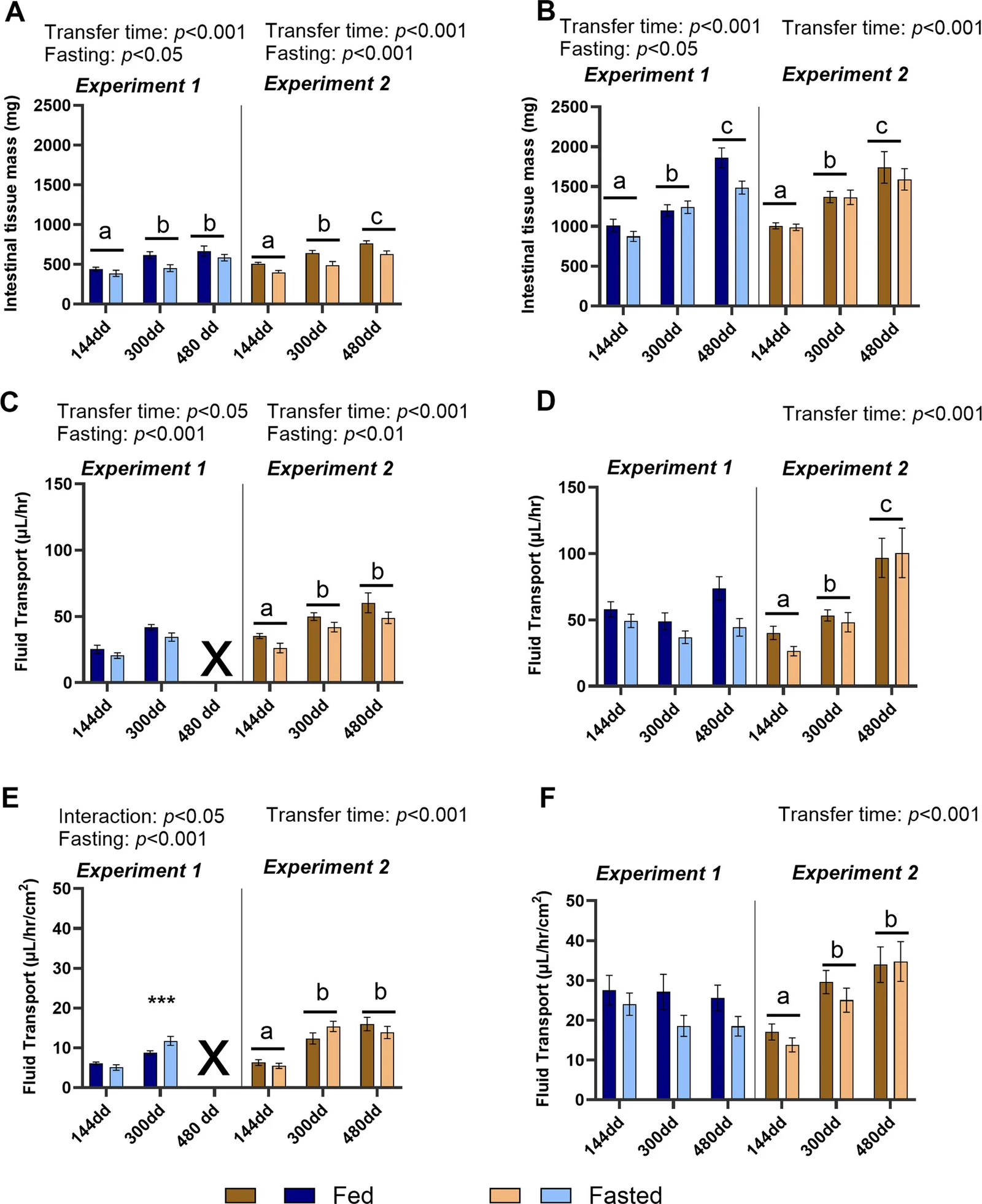
Intestinal tissue mass (mg), intestinal fluid transport capacity per intestine, and per area (Jv) of the intestine are presented for FW smolts (**A**, **C**, **E**) and SW post-smolts (**B**, **D**, **F**). Experiment 1 denotes fish fed a control diet (blue bars) during smoltification, whereas Experiment 2 denotes fish fed a high-lipid diet (brown bars) during smoltification. **B**, **D**, and **E** graphs indicate post-smolts fed the same diet in SW, with colours indicating their dietary history during smoltification: control diet (blue) and high-lipid diet (brown). Results are shown for the different transfer times, with fed and fasted groups indicated in the legend. Different lowercase letters indicate significant differences between transfer groups. For transfer time × fasting interactions, asterisks (*) denote significant differences between fed and fasted subgroups within the same transfer group. The X denotes no available data for that transfer group. Results are presented as mean ± SEM. (*p < 0.05, ** *p* < 0.01, *** *p* < 0.001) and *n* = 9 fed and *n* = 9 fasted fish per transfer time were used

FW fish transferred both at 300 dd and 480 dd displayed higher intestinal mass compared to the fish transferred at 144 dd (*p* < 0.001, Fig. 4A). After 6 weeks in SW, the 480 dd transfer group had the highest intestinal mass, followed by the 300 dd and 144 dd transfer groups, respectively (*p* < 0.001, Fig. 4B).

The fasted fish in general had lower intestinal mass compared to the fed fish, both in FW (*p* < 0.05, Fig. 4A) and in SW (*p* < 0.05, Fig. 4B).

#### Intestinal somatic index (ISI)

After 6 weeks in SW, there was a significant transfer effect (*p* < 0.05), and post hoc test showed a tendency that fish transferred at 144 dd had a smaller ISI compared to 480 dd. Fasting did not significantly influence the ISI in post-smolts.

#### Intestinal fluid transport capacity

Intestinal fluid transport per intestine (µL/hr) in FW was higher in the 300 dd transfer group compared to the fish transferred at 144 dd (*p* < 0.05, Fig. 4C). Fluid transport capacity was not analysed in the 480 dd transfer group in FW due to a technical error. In post-smolts, there was no significant difference among transfer groups in fluid transport capacity (Fig. 4D). Overall, post-smolts in SW had a higher tendency for intestinal fluid transport capacity than the FW fish. Fasted fish showed a general reduction in fluid uptake compared to the fed fish in FW (*p* < 0.001, Fig. 4C). No effect of fasting on fluid uptake per intestine was observed in SW (Fig. 4D).

Jv (µL/hr/cm^2^) in FW was significantly higher in the group transferred at 300 dd compared to the 144 dd transfer group (interaction, *p* < 0.05; Fig. 4E). No significant difference between transfer time groups was observed at 6 weeks in SW (Fig. 4F). Overall, post-smolts in SW had a higher tendency for Jv than the FW fish. Fasted fish transferred at 300 dd in FW had significantly higher Jv compared to the fed fish in the same group (interaction, *p* < 0.05; Fig. 4E), while fasting did not significantly affect Jv in SW post-smolts (Fig. 4F).

**Fig. 5:**
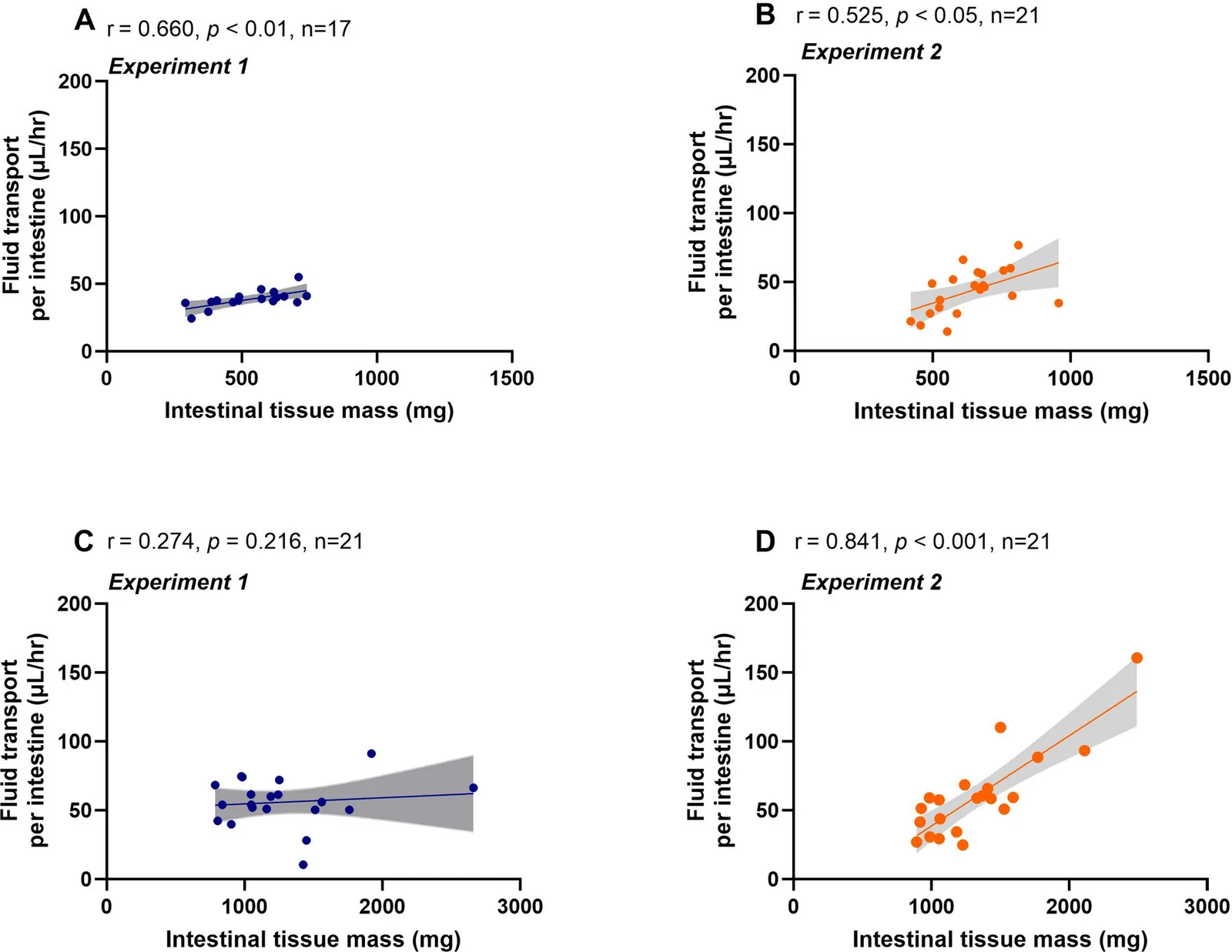
Correlation between intestinal tissue mass and fluid transport capacity in FW fish (**A** and **B**) and SW post-smolts (**C** and **D**). Experiment 1 (blue) denotes the control diet during smoltification, and Experiment 2 (brown) denotes the high-lipid diet during smoltification. **C** and **D** denote post-smolts fed the same diet in SW, with colours indicating feeding history during smoltification: control diet (blue) and high-lipid diet (brown)

The correlation between intestinal tissue mass and fluid transport capacity revealed that intestinal tissue mass was positively correlated with intestinal fluid transport capacity in FW (*p* < 0.05, Fig. 5A) but not in SW (Fig. 5C).

#### Intestinal transport and barrier functions

**Fig. 6:**
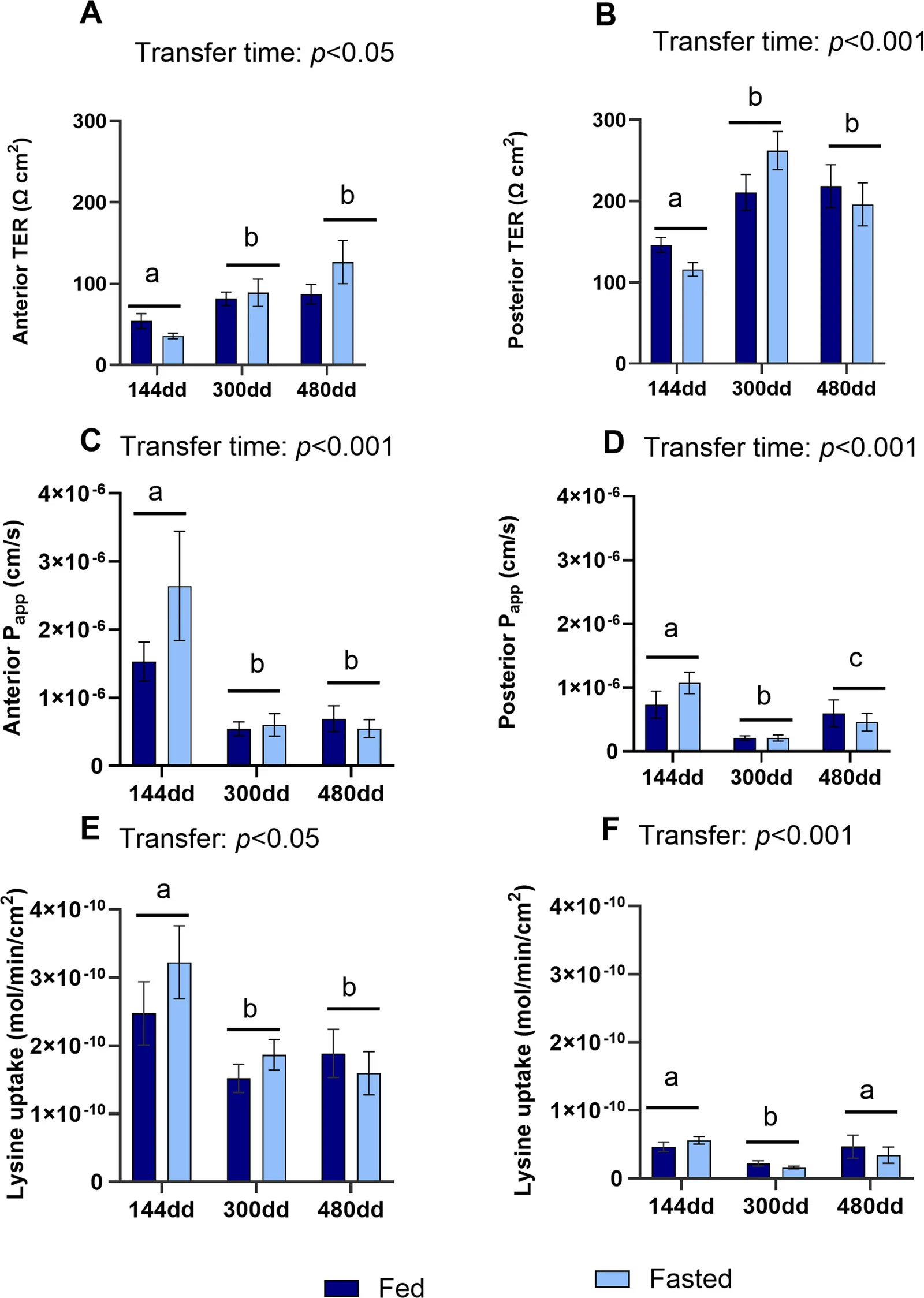
Transepithelial resistance, TER (Ω cm^2^), apparent permeability, P_app_ (cms^−^^1^), and lysine uptake (mol min^−1^cm^−2^) for control diet received post-smolts for both anterior (**A**, **C**, **E**) and posterior (**B**, **D**, **F**) intestinal regions. Results are shown for the different transfer groups, with fed and fasted groups indicated in the legend. Different lowercase letters indicate significant differences between transfer groups. For transfer time × fasting interactions, asterisks (*) denote significant differences between fed and fasted subgroups within the same transfer group.

In SW, both 300 dd and 480 dd transfer groups had higher TER in the anterior intestine compared to the fish transferred at 144 dd (*p* < 0.05, Fig. 6A). The same pattern was also observed in the posterior intestine (*p* < 0.001, Fig. 6B). *P*_*app*_ showed higher values for the 144 dd transfer group compared to the 300 dd and 480 dd groups in both anterior (*p* < 0.001, Fig. 6C) and posterior intestines (*p* < 0.001, Fig. 6D). A similar pattern was also observed in the L-lysine uptake in the anterior intestine (*p* < 0.05, Fig. 6E). In the posterior intestine, both 144 and 480 dd transfer groups had higher L-lysine uptake compared to the 300 dd transfer group (*p* < 0.001, Fig. 6F). Considering the numerical values, the posterior intestines of all transfer groups had a tendency for higher TER but lower *P*_*app*_ and lysine uptake compared to the anterior intestines. None of these parameters was significantly affected by fasting (Fig. 6A–F).

### Experiment 2—fasting after feeding a high-lipid diet and SW transfer time

#### NKA activity

Both 300 dd and 480 dd transfer groups had higher NKA activity in FW compared to the 144 dd transfer group in both anterior and posterior intestine (*p* < 0.001, Fig. 3A, B). Fasted fish had generally higher intestinal NKA activity in FW, both in anterior (*p* < 0.05, Fig. 3A) and posterior intestines (*p* < 0.001, Fig. 3B) compared to the fed fish.

In SW, anterior intestinal NKA activity was significantly higher in the 480 dd transfer group compared to the other two transfer groups (*p* < 0.001, Fig. 3C). Posterior intestinal NKA activity was high at both 300 dd and 480 dd compared to 144 dd (*p* < 0.001, Fig. 3D). No significant effects of fasting on NKA activity were observed in SW (Fig. 3C, D).

#### Intestinal tissue mass

In both FW and SW, 480 dd transfer group had the highest intestinal tissue mass, followed by 300 dd and 144 dd, respectively (*p* < 0.001; Fig. 4A and B). The fasted fish in FW had lower intestinal tissue mass in general compared to the fed fish (*p* < 0.001, Fig. 4A). In SW, the intestinal tissue mass was not significantly affected by fasting (Fig. 4B).

#### Intestinal somatic index (ISI)

ISI was not significantly affected by transfer time or fasting in SW post-smolts after 6 weeks in SW.

#### Intestinal fluid transport capacity

The fluid uptake per intestine (µL/hr) was higher in both 300 dd and 480 dd transfer groups in FW compared to the 144 dd group (*p* < 0.001, Fig. 4C). In SW post-smolts, intestinal fluid uptake was higher in the group transferred at 480 dd than in the other two transfer groups (*p* < 0.001; Fig. 4D). Overall, post-smolts in SW had a higher tendency for intestinal fluid transport capacity than the FW fish. Fasted fish had generally lower fluid uptake per intestine compared to the fed fish in the same transfer group in FW (*p* < 0.01, Fig. 4C), but no significant effect of fasting was observed in SW (Fig. 4D).

Jv (µL/hr/cm^2^) was higher at both 300 dd and 480 dd compared to the 144 dd transfer group in FW and in SW (*p* < 0.001, Fig. 4E, F) but was not affected by fasting (Fig. 4E, F). In general, post-smolts in SW had a higher tendency for Jv than the FW fish.

There was a significant correlation between intestinal tissue mass and intestinal fluid transport capacity in both FW (*p* < 0.05, Fig. 5B) and SW (*p* < 0.001, Fig. 5D).

### Fish survival

For both experiment 1 and 2, no fish mortality was observed during FW or SW up to 6 weeks, resulting 100% fish survival during the study period.

## Discussion

The present study examined intestinal development of hypo-osmoregulatory ability during smoltification using NKA enzyme activity, intestinal tissue mass and fluid transport capacity in response to different SW transfer times, dietary lipid levels, and short-term fasting prior to SW transfer.

The results show that the intestine grows during smoltification, as observed in both experiments, with an increase in tissue mass over time spent in FW. The increase in intestinal mass probably reflects the generation of all tissue types in the intestinal wall, including increased epithelial surface area (Duarte et al. 2023). For instance, Duarte et al. (2023) observed increased absorptive perimeter and number of villi in the intestine in FW smolts. An increase in epithelial surface area would, by itself, contribute to increased transport capacity since the epithelium is responsible for all transport across the intestine. This is supported by the positive relationship between intestinal tissue mass and intestinal fluid transport capacity per intestine observed for both control and high-lipid diets (Fig. 5A, B). In addition, the gradual increase in NKA activity over time in FW, observed in both control and high-lipid diet-fed fish, would also contribute to the increase in fluid transport capacity (Loretz 1995). Our results for FW fish indicate that fluid transport capacity, driven by increases in intestinal mass and surface area and NKA enzyme activity, remains elevated at least until 480 dd. The present results agree with previous studies that pointed out transferring to SW later in the smolt window would be beneficial for enhancing osmoregulatory capacity (Takvam et al. 2024; Silva-Marrero et al. 2025; de Fonseka et al. 2026).

The results of intestinal development in FW further support the idea that 480 dd represented a favourable transfer time in both dietary groups. However, the high-lipid diet appeared to strengthen the development of hypo-osmoregulatory capacity a little earlier than 480 dd. This was supported by the significant increase of fluid transport capacity concurrent with NKA activity already at 300 dd. In contrast, the control diet-fed fish showed increased NKA activity at 480 dd, and it is likely that this would be followed by elevated transport capacity. Unfortunately, the fluid transport capacity measurements at 480 dd in the control diet were lost due to a technical issue. Nevertheless, the interpretation is supported by previous studies reporting increased Jv with increased NKA activity during smoltification (Veillette et al. 1993; Sundell et al. 2003). It should also be noted that 480 dd may not represent the peak development of intestinal fluid uptake, as intestinal NKA activity increased beyond this point, at least when fish were fed a commercial diet similar to the control diet used in this experiment (Sundell et al. 2003; de Fonseka et al. 2026). Similarly, kidney NKA activity has been reported to continue increasing after the peak in gill NKA activity at 300–400 dd (Takvam et al. 2021, 2024).

In this study, intestinal fluid transport capacity was used as a proxy for hypo-osmoregulatory capacity. The established method for calculating Jv is per area of the intestine (µL/hr/cm^2^) (Collie and Bern 1982; Veillette et al. 1993, 1995; Sundell et al. 2003). Here we also report fluid transport capacity per intestine (µL/hr). The main argument for this is that when assessing the transport capacity of the entire intestine, the results reflect the total fluid uptake capacity of these intestinal regions that is available for the fish. On the other hand, Jv reflects transport efficiency regardless of intestinal size (Veillette et al. 1993, 1995; Sundell et al. 2003). When examining hypo-osmoregulatory development, it is important to understand the total capacity that fish have developed, rather than how much fluid they can transport in an area of the intestine (Jv). Nevertheless, Jv provides valuable complementary information on physiological compensation and allows for comparisons across studies because it is independent of fish size.

In SW post-smolts, the elevated intestinal fluid transport capacity per intestine observed at 480 dd likely reflects the combined effect of increased intestinal size and elevated NKA activity at this sampling point relative to the earlier transfer groups. This response was most pronounced in fish fed the high-lipid diet, although fish fed the control diet showed a tendency relative to the earlier transfer groups. Such increases are expected in SW-acclimated fish, as the transition to SW requires increased drinking and intestinal water absorption (Smith 1930; Veillette et al. 1993). However, it is important to consider that since there was a 2-week time difference between each transfer group, the later groups would naturally be expected to have a higher transport capacity due to continued intestinal growth. Further, the finding that intestinal size was poorly correlated to fluid transport capacity in post-smolts fed the control diet during smoltification was surprising. One possible explanation behind these findings is that the epithelial area may not be developing as fast even though the intestine grows in mass. In a previous experiment, de Fonseka et al. showed that smolt size increased significantly with the time spent in FW (de Fonseka et al. 2026). The present findings extend this understanding by suggesting that prolonged FW residence not only increases fish size but also increases the intestinal size over time which provides the smolt with higher intestinal transport capacity at the time of transfer to SW. Both of these factors are beneficial for the fish in SW since larger fish have a lower surface area-to-volume ratio which supports the ability to osmoregulate (Parry 1960; Duston 1994) and reduces osmoregulatory cost (Ytrestøyl et al. 2023) while a bigger intestine provides conditions for increased fluid uptake needed in SW. The importance of the additional time provided for smolt development in FW in the 480 dd transfer group was apparent in the weight gain during the first 6 weeks in SW, where the 480 dd transfer group had 44.4% and 30.1% higher weight gain compared to the 144dd and 300 dd transfer groups, respectively and smolts transferred at 480 dd outgrew earlier transfer groups for up to 5 months in SW in both diet groups when sampled on the same day, indicating a sustained effect of transfer time on fish growth (de Fonseka et al 2026). Thus, our results support that intestinal hypo-osmoregulatory development is a key determinant of SW growth and should be considered when determining transfer timing, particularly given the diverse smolt production protocols used today (Lysfjord et al. 2004; Striberny et al. 2021; Pino Martinez et al. 2021).

Even if the intestines of the fish transferred at 144 dd were the smallest and had the lower NKA activity compared to 480 dd transfer group after 6 weeks in SW, Jv was in the same range in all transfer groups. A similar effect was reported by Duarte et al. (2023), who observed increased intestinal thickness in the posterior intestine of post-smolts after SW transfer. This may reflect that the fish in the 144 dd group experience higher osmoregulatory stress due to their relatively larger body surface area-to-volume ratio compared to the larger fish (Parry 1960; Duston 1994).

Only the 144 dd transfer group fed the control diet during smoltification exhibited reduced intestinal barrier function compared to the other two transfer groups as reflected by lower TER and increased P_app_ for mannitol in both intestinal regions. Electrophysiological characteristics of the epithelium are commonly used to assess intestinal transport capacity through the ion-coupled active transport and changes in epithelium permeability (Sundell and Sundh 2012). Increased paracellular permeability is also a known secondary stress response in vertebrates (Söderholm and Perdue 2001; Knudsen et al. 2008; Sundh et al. 2010; Tabler et al. 2020) suggesting that this group is subjected to stress. Further, this group also tended to have a lower ISI after 6 weeks in SW showing that the intestinal growth was negatively affected by the early transfer. The relatively smaller intestine in relation to fish size provides poorer conditions for intestinal transport. In addition, the smaller fish size makes these fish more sensitive to osmoregulatory stress due to the lower surface area-to-volume ratio. To sustain growth and osmoregulation, the fish would need to compensate. Interestingly, the 144 dd group also indicated elevated lysine uptake in the proximal intestine suggesting compensatory increase in nutrient transport. This group also seems to sustain fairly high fluid transport capacity as no significant differences were observed between groups. However, this compensation does not appear to be enough since this group had a significantly lower weight gain from FW to 6 weeks in SW compared with the other two transfer groups and lower body weight up to 5 months after SW transfer (de Fonseka et al 2026). Together, these findings suggest that fish fed the control diet during smoltification and transferred to SW at 144 dd experienced elevated physiological stress and impaired intestinal barrier function during the early SW period.

Before SW transfer, a 4-day fasting period was performed to mimic the standard pre-SW transfer routine in Atlantic salmon farming. There was a clear pattern in fish fed both diets showing that fasting reduced intestinal tissue mass in FW fish across all transfer times, independent of smolt development. Our findings of intestinal tissue mass reduction are consistent with previous studies reported in Atlantic salmon post-smolts (Krogdahl and Bakke-McKellep, 2005) and other FW teleosts, including rainbow trout (Simpkins et al. 2003) and common carp (Gas and Noailliac-Depeyre 1976). Consistent with reduced intestinal tissue, we observed that fluid uptake per intestine was also lower in fasted than in fed FW fish. In farmed Atlantic salmon, gut evacuation is typically complete within 2 days of fasting at 6–18 °C (Aas et al. 2017), whereas a longer fasting time induces structural changes (Zaldúa and Naya 2014) that suppress intestinal function to save energy while food is absent (Cant et al. 1996; Wang et al. 2006). Together, these findings indicate that even short fasting periods, such as 4 days, can adversely affect intestinal structure in sensitive life stages and compromise osmoregulatory capacity. This study focused on the effects of fasting on Atlantic salmon smolts, whereas previous studies have exclusively examined post-smolts (Olsen et al. 2002; Hvas et al. 2021; 2022). Overall, the present results suggest that fasting may impair intestinal development associated with smoltification and these effects may exacerbate under commercial aquaculture conditions, where smolts in relation to SW transfer are routinely exposed to additional stressors such as crowding, chasing, and transport.

After 6 weeks in SW, the 4 days of fasting prior to SW transfer had sustained negative effects in the fish fed the control diet. This was evidenced by the reduction in intestinal tissue mass concurrent with a tendency of lower fluid uptake capacity per intestine. Thus, 6 weeks of re-feeding were insufficient to fully restore intestinal tissue mass in the fasted fish. Integrating these findings of intestinal physiology with previously reported growth data from the same study it appears that recovery from fasting depends on the degree of hypo-osmoregulatory development at the time of SW transfer. Fish transferred to SW at 480 dd recovered faster and grew better, whereas fasted fish transferred at 144 dd had impaired growth up to 5 months in SW (de Fonseka et al. 2026). Overall, our results suggest that short-term fasting before SW transfer can substantially impair the intestine and its transport capacity for at least 6 weeks after SW transfer. Importantly, fish fed a high-lipid diet during smoltification recovered from fasting after 6 weeks with no lasting effects on growth, not even in the 144 dd group. Although the two diets in FW were not statistically compared, fish growth was within the same numerical range across sampling points indicating the extra energy from high-lipid diet did not primarily allocate to somatic growth (de Fonseka et al. 2026). Thus, 5% extra energy in lipids appears to protect the intestine from the negative effects of fasting. It is implied that the extra lipids provide the enterocytes with energy stores that can be used during fasting to sustain the intestinal tissue mass. In support of this, fasting had no negative effects on intestinal tissue mass at any transfer time in the high-lipid group.

## Conclusion

In this study, we demonstrated that, for both the control and high-lipid diets, delaying SW transfer later in the smolt window associates with more developed intestines during smoltification. This improves the intestinal fluid transport capacity and increases intestinal barrier function of the fish during the critical first months in SW. Fish fed the control diet and transferred at 144 dd showed signs of poor SW adaptation. Fish fed a high-lipid diet (5% additional fat) accelerated the development of hypo-osmoregulatory ability. Fasting before transfer reduced intestinal tissue mass and compromised total fluid uptake capacity; however, the high-lipid diet protected the fish from the negative effects of fasting. Together, these findings underscore the significance of evaluating intestinal fluid transport capacity as a key indicator of smolt SW readiness, which is crucial for producing robust smolts in modern aquaculture. Further research is required to determine the minimum fasting duration that can be applied to smolts without causing significant adverse effects, as well as the length of refeeding necessary for full intestinal recovery. Moreover, how to incorporate high-lipid diets for boosting smolt development and osmoregulatory capacity is worth exploring.

## Data Availability

The data supporting this study’s findings are available upon request.
